# The Influence of Marriage on Mortality in France

**Published:** 1859-02

**Authors:** 


### SELECTIONS.

From the Virginia Medical Journal.

*The Influence of Marriage on Mortality in France.* By the Registrar General of Great Britain.

A PAPER was read by Dr William Farr at the Liverpool congress of social science, on the influence of marriage on the health of the French population, in which several facts were presented of the highest interest, proving that marriage is the unit of the social state—in other words, favorable alike to health and longevity. As we have no statistics from which to treat this great question in relation to English lives, the facts presented in reference to France have a peculiar interest, and will not fail to command attention in this country. We refer to the analysis presented by Dr. Farr, not as giving a substitute for this kind of information as to our own country; but in the hope we shall not always be left destitute of similar returns, through the important department of the public service with which the author of the paper is connected.

The facts and figures now given for the first time are based on returns extending over the whole of France, and among all classes of the people, thus exceeding greatly in value any thing ever before produced by writers of that country, who have directed their studies to the relative value of single and married life. It will be remembered that Deparcieux, in the middle of the last century, investigated the relative mortality of monks and nuns in France, contrasting their lives with those of Tontine annuitants consisting partly of married, and partly of unmarried. His enquiry was thus limited as to area and classes, insted of being, as now, extended over all persons in all parts of France. The result he obtained was, that from the age of 20 to 40, the mortality of the *religieux* was low, and after the age of 40 it was high, as compared with the rate of mortality amoung the Tontine annuitants. The statistics now furnished give us the effect of the conjugal condition on the life of the whole population, minors, of course, being excluded.

In reference to the conjugal relation, the more than thirtysix millions included in the French census of 1851 are divided into three classes:

1. The married—consisting of 6,986,223 husbands, and 6,948, 823 wives = 13,935,046 married persons.

2 The celibates, or persons who never married—bachelors, 4,014, 105, and spinsters, 4,549,044 = 8,564, 049.

3. The widowed—widowers, 836,509; widows, 1, 687,583 = 2, 524,092.

Dealing then with these nearly fourteen millions of married persons, the author notices, first the case of husbands and wives under the age of 20, marriage being legal in France for men at 18, and woman at 15. In this early age the mortality was excessive. Twice as many wives died under the age of 20 as died in the same number of unmarried woman; while the mortality was also found to be much higher among married than the unmarried men. Among the married women, the rate of mortality was 14.0 in 1,000, and among the spinsters it was only 8.0. Among the married men it was 29.0 in 1000, and among the unmarried it was 7.0. This result confirms the opinion of the evil consequences in many cases of marriage under the age of 20, before the growth of the individual man or woman is completed.

In the next twenty years, 20—40, wives show a rate of mortality half as high as again as husbands; the mortality of wives from 20—30 being at the rate of 9.3 in 1000, and from 30—40 at the rate of 9.1, while the mortality of husbands is 6.5 in the first interval, and 7.1 in the second. The excess on the female side is obviously ascribable to the perils of child-bearing, aggravated by unskillful female assistance. From 40—50 the mortality of husbands (18.3) is slightly higher than of wives, and continues higher in the subsequent ages, though the difference in favor of wives is not considerable, as will be seen in the following table:

| Age. | Husbands. | Wives. |
|---|---|---|
| 50—60 | 18.3 | 16.3 |
| 60—70 | 35.4 | 35.4 |
| 70—80 | 88.6 | 84.9 |
| 80—90 | 183.6 | 180.4 |

Turning now to the celibates. It has already been observed at the younger ages, under 20, that the rate of mortality is lower in both the sexes than among the married, being in males 6.0, and in females 7.1 in 1000.

Taking the ages from 20—60, unmarried men show a much higher rate of mortality than unmarried women; being in the first ten years

(20—30) in the ratio of 11.3 to 8.7. This excess is partly attributable to deaths in Algeria, and in the *casernes* at home; but these elements do not interfere in subsequent stages to any considerable extent. From 30—40, deaths among men were 12.4, among women 10.3. From 40—50, men 17.7; women 13.8. From 50—60, men 29.5; women 23.5. From 60 upwards, the rate of mortality was nearly equal in the sexes.

Comparing the married with the unmarried, it is found that between the ages of 20 and 25, maidens have the advantage, the difference being considerable. Of 1000 females the annual deaths among the married were 9.8, and among the unmarried 8.5. At the ages of from 25—30 the mortality of the unmarried is slightly in excess, being 9.2 to 9.0. At the ages 30—40 the mortality of wives was 9.1, while that of unmarried women was 10 3. After the age of 40, married women show a much lower rate of mortality than the unmarried, the rates being from 40—50 married, 10.0; unmarried 13.8. From 50—60, married 16 3; unmarried 23.5; and from 60 upwards, married 35.4; unmarried 49.8.

The contrast between the bachelors and married men is very remarkable, in the earlier ages being in favor of bachelors, and the more advanced against them.

The widowed are found to have a higher rate of mortality than the celibate, who it has been shown have a greater mortality than the married. At all the ages under 40 the mortality of widows is higher than the mortality of unmarried women, while at the earlier ages the rate is double. Advancing from the age of 40 the mortality of widows is lower than that of spinsters, and at all ages the mortality of widows is greater than that of wives. Among widowers it is found that there is a heavy rate of mortality under the age of 30, and even under 40, while afterwards they die more rapidly than not only the husbands, but even the bachelors.

Thus, it results from this novel enquiry that marriage is a healthy state, the single being in danger of wreck, where those united in matrimony often survive the storm.

